# Coding-Complete Genome Sequences of an Iteradensovirus and an Alphapermutotetra-Like Virus Identified from the Pine Processionary Moth (Thaumetopoea pityocampa) in Portugal

**DOI:** 10.1128/MRA.01163-20

**Published:** 2021-01-07

**Authors:** Franck Dorkeld, Réjane Streiff, Carole Kerdelhué, Mylène Ogliastro

**Affiliations:** a CBGP, INRAE CIRAD IRD Montpellier SupAgro Université de Montpellier, Montpellier, France; b DGIMI, INRAE Université de Montpellier, Montpellier, France; KU Leuven

## Abstract

The coding-complete genome sequences of an iteradensovirus (family *Parvoviridae*) and an alphapermutotetra-like virus (family *Permutotetraviridae*) were discovered from transcriptomic data sets obtained from Thaumetopoea pityocampa larvae collected in Portugal. Each of the coding-complete genome sequences of these viruses contains three main open reading frames (ORFs).

## Abstract (precis)

The coding-complete nucleotide sequences of an iteradensovirus (family *Parvoviridae*) and an alphapermutotetra-like virus (family *Permutotetraviridae*) were discovered from transcriptomic data sets obtained from Thaumetopoea pityocampa larvae collected in Portugal. Each of the coding-complete genome sequences of these viruses contain three main open reading frames (ORFs).

## ANNOUNCEMENT

Pine processionary moth (PPM) caterpillars are a major threat to conifer forests in Mediterranean regions (1). They live in silken nests in pine tree branches that they leave to travel to the ground in a characteristic head-to-tail procession. The caterpillars are covered with urticating hairs that can pose major allergic reactions to humans and domestic animals in high densities. Pest management formerly relied on aerial spraying of Bacillus thuringiensis strain kurstaki; this method is now strongly limited in Europe (2).

Entomopathogenic viruses were considered to control PPM in the 1950s but never developed (3). Today, metagenomic approaches represent new opportunities to explore virus diversity in PPM (4).

Here, we describe the complete coding genome sequences of two viruses infecting Lepidoptera found in PPM larvae collected for a population survey in Portugal. Total RNAs were extracted from 2 to 5 third-instar caterpillars with TRIzol reagent, pooled in equimolar amounts. Libraries were constructed using the TruSeq stranded mRNA prep kit (Illumina) with an insert size of 450 bp and sequenced in two lanes of paired-end (PE) 2 × 100-bp reads on the HiSeq 2000 platform (5). We obtained 31,909,769 reads that were paired-end trimmed (Trimmomatic v.0.33) (6) and assembled into contigs (Trinity v.2.0.2) (7). Default parameters were used for all software unless otherwise specified. Viral reads were identified by subjecting contigs consecutively to BLASTX (E value, 1.e-10) against the viral RefSeq and BLASTN nonredundant (nr) databases (NCBI). We found 436,806 viral reads, representing 1.3% of the total reads. Taxonomic sequence assignment identified the full coding genome sequences of an iteradensovirus, a single-stranded linear DNA virus (family *Parvoviridae*), and an alphapermutotetra-like virus, a single positive-strand RNA virus (family *Permutotetraviridae*).

**FIG 1:**
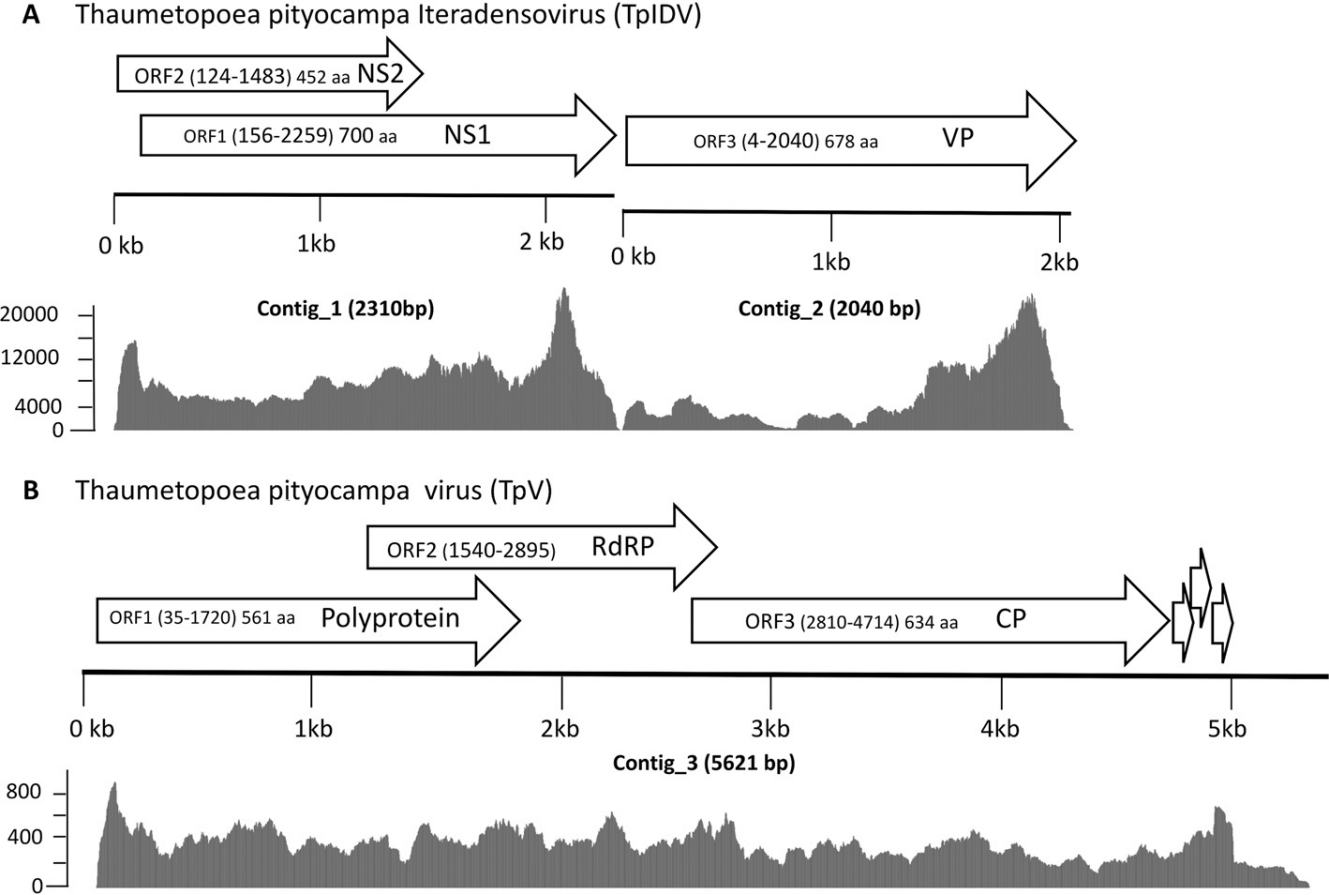
Genomic organization, matching reads, and coverage of *Thaumetopoea pityocampa* iteradensovirus (TpIDV) contigs (assembled from 233,972 and 176,824 reads, respectively) (A) and a *Thaumetopoea pityocampa* virus (TpV) contig, made from the assembly of 20,260 reads (B). Complete major open reading frames (ORFs) predicted are represented by arrows indicating putative proteins. NS, nonstructural protein; VP, capsid protein; RdRP, RNA-dependent RNA polymerase; CP, capsid protein. Three putative small ORFs are represented at the 3′ end (nucleotides 4758 to 4961). Matching reads mapped to the virus genomes are represented by the gray areas, with the scale representing the coverage. The solid lines represent the contigs.

The Thaumetopoea pityocampa iteradensovirus (TpIDV) has two contigs assembled from 233,972 and 176,824 reads (mean coverage, 6,138×), representing 94% of the total viral reads (Fig. 1A). Contig-1 (2,310 bp; GC content, 38.2%) has two predicted overlapping open reading frames (ORF1 and ORF2), corresponding to two nonstructural proteins (NS1 and NS2); Contig_2 (2,033 bp; GC content, 37.3%) has one predicted ORF (ORF3), corresponding to a structural protein (VP). The VP has a consensus phospholipase A2 motif (position 9 to 96), like many parvoviruses (8). Amino acid (aa) sequences are 98 to 95% identical to those of an unclassified iteradensovirus discovered recently from bat guano and ∼89% identical to those of the Danaus plexippus *plexippus* iteradensovirus (GenBank accession numbers MG963176.1 and KF963252, respectively) (9).

The *Thaumetopoea pityocampa* virus (TpV) is made of one contig (5,621 bp; GC content, 46.3%) assembled from 20,260 reads (mean coverage, 306×), which represent 4.6% of the total viral reads (Fig. 1B). Three main overlapping ORFs were predicted, displaying ∼30% amino acid identity with the protein of an unclassified fish virus (ORF1; GenBank accession number YP_009361866), 34% amino acid identity with the putative RNA-dependent RNA polymerase (RdRP) from the Hubei leech virus 1 (ORF2; GenBank accession number NC_032925.1), and 49% amino acid identity with the capsid protein (CP) of the Thosea asigna virus (ORF3; GenBank accession number NC_043232), the type species of the genus *Alphapermutotetravirus* (10). Putative small ORFs (position 4758 to 5275) and RNA secondary structures (position 5300 to 5626; >50% prediction: RNAstructure Web server, http://rna.urmc.rochester.edu/RNAstructureWeb) at the 3′ end might correspond to minor CP(s) and pseudoknots described in this virus family (11).

### Data availability.

The GenBank accession numbers are MT796426 and MT796427 for *Thaumetopoea pityocampa* iteradensovirus and MT796428 for *Thaumetopoea pityocampa* virus. The reads were deposited in the Sequence Read Archive (SRA) NCBI database under BioProject accession number PRJNA663237.
